# Supplementary material for: A complex of BRCA2 and PP2A-B56 is required for DNA repair by homologous recombination
Source: Nat Commun. 2021 Sep 30;12:5748. doi: 10.1038/s41467-021-26079-0 (PMC8484605; doi:10.1038/s41467-021-26079-0)
Supplement: Supplementary file 1 — Supplementary information [file 41467_2021_26079_MOESM1_ESM.pdf]

Supplementary information

A complex of BRCA2 and PP2A-B56 is required for DNA repair by homologous recombination.

Ambjørn *et al*.

Content of supplementary information:

Supplementary Figure 1. Data related to Figure 1.

Supplementary Figure 2. Data related to Figure 2.

Supplementary Figure 3. Data related to Figure 2.

Supplementary Figure 4. Data related to Figure 3.

Supplementary Figure 5. Data related to Figure 3.

Supplementary Figure 6. Data related to Figure 4.

Supplementary Figure 7. Uncropped Western blot and gel images.

Supplementary Table 1. DNA oligos.

Supplementary Table 2. Isothermal titration calorimetry data.

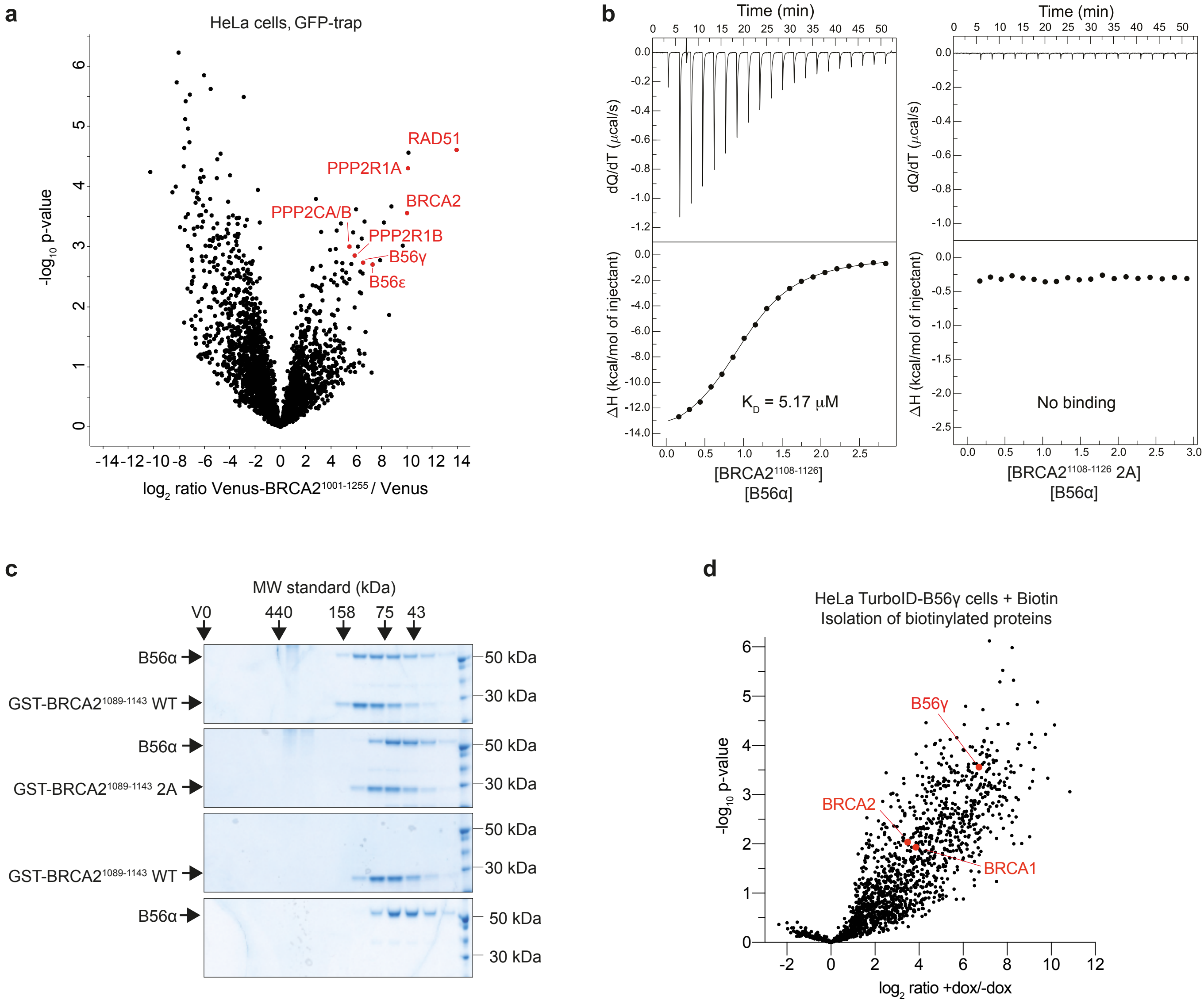

Supplementary Figure 1. Data related to Figure 1.

a. Volcano plot showing proteins co-purifying with Venus or Venus-BRCA2$^{1001\text{-}1255}$. HeLa cells were transiently transfected with constructs of Venus or Venus-BRCA2$^{1001\text{-}1255}$, synchronized to S phase with a thymidine block, released for 2 hours, and then treated for 2 hours with 100 nM CPT prior to cell harvest, GFP-trap immunoprecipitation, and mass spectrometry analysis. Statistical analysis was carried out by two-tailed Student's t-test of three technical replicates. **b.** Isothermal titration calorimetry binding curves for the interaction between BRCA2$^{1108\text{-}1126}$ WT or 2A peptides and B56α. **c.** Colloidal stained gel showing the gel filtration chromatography of B56α with GST-BRCA2$^{1089\text{-}1143}$ WT or 2A representative of one experiment. **d.** Volcano plot of a screen for B56γ proximity partners using biotin proximity labelling coupled to mass spectrometry. HeLa Flp-In T-REx cells encoding doxycycline-inducible TurboID-B56γ were induced (+dox) or not induced (-dox) alongside synchronization to S phase with a thymidine block. Cells were released from the block for 2 hours in presence of 100 nM CPT, and 50 mM biotin was added 30 minutes before harvest. Biotinylated proteins were purified and analyzed by mass spectrometry. Statistical analysis was carried out by two-tailed Student's t-test of three independent experiments.

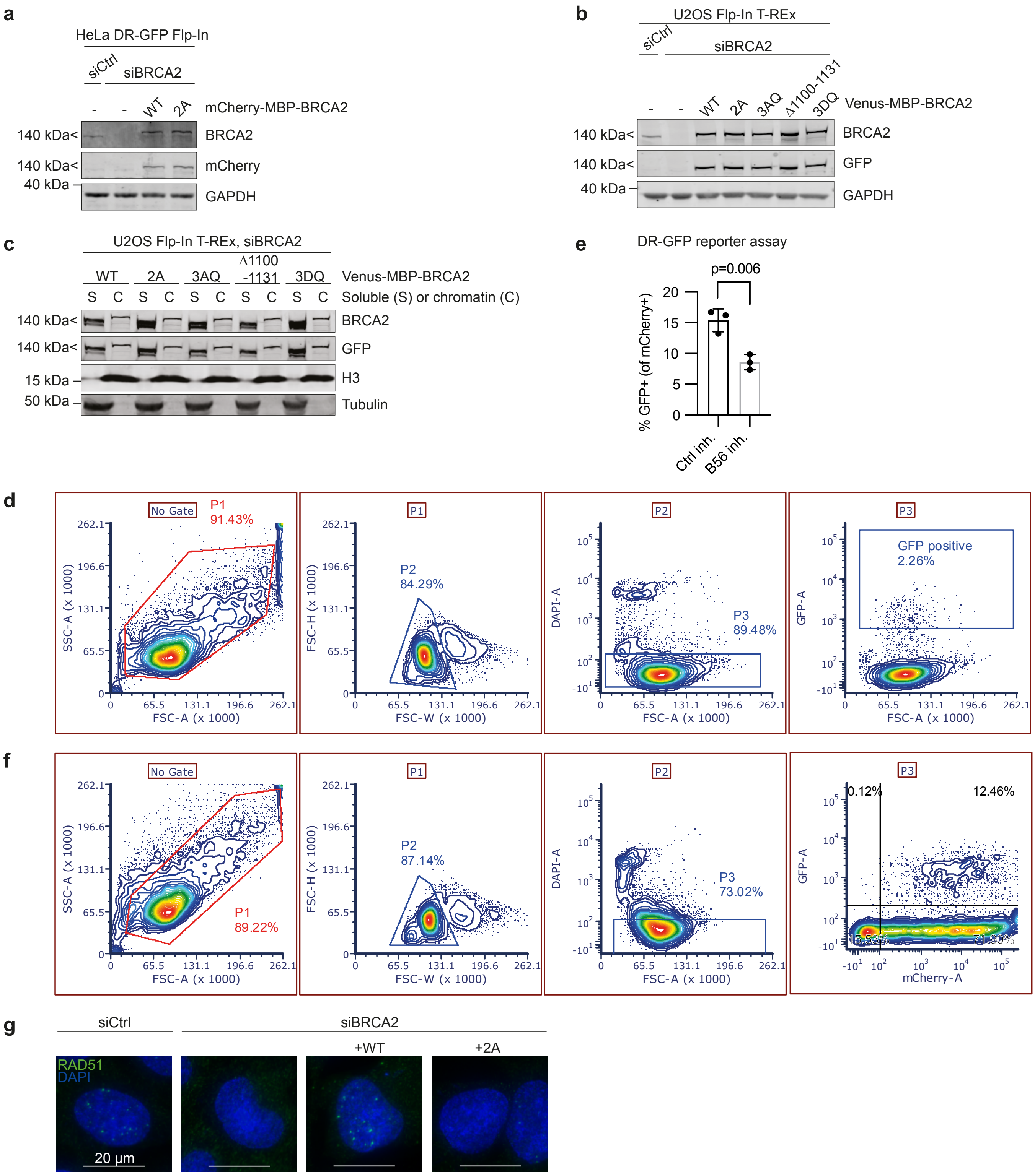

Supplementary Figure 2. Data related to Figure 2.

a. Western blot of cell extracts from HeLa DR-GFP Flp-In parental cells or stably expressing siRNA resistant mCherry-MBP-BRCA2 WT or 2A cDNAs after transfection with Ctrl or BRCA2 siRNA. Representative of two independent experiments. **b.** Western blot of cell extracts from U2OS Flp-In T-REx parental cells or stably expressing the indicated siRNA resistant Venus-MBP-BRCA2 cDNAs after transfection with Ctrl or BRCA2 siRNA. Representative of two independent experiments. **c.** Western blot of fractionated chromatin from U2OS Flp-In T-REx cells stably expressing the indicated siRNA resistant Venus-MBP-BRCA2 cDNAs after BRCA2 siRNA transfection representative of one experiment. **d.** Example of flow cytometry gating strategy for Fig. 2a. **e**. Percentage of GFP positive (HR completed) HeLa DR-GFP Flp-In cells within the mCherry positive population of cells transfected with an I-SceI-encoding plasmid alongside an mCherry-tagged inhibitor of PP2A-B56 binding to LxxIxE motifs (B56 inh.) or a control inhibitor (Ctrl inh.). Background values (without I-SceI) were subtracted. Data are presented as means +/- standard deviations, and individual data points are indicated for three independent experiments. Two-tailed Student’s t-test was performed, p=0.006. **f.** Example of flow cytometry gating strategy for Supplementary Fig. 2e. **g.** Representative immunofluorescence microcopy images of RAD51 foci from Fig. 2f.

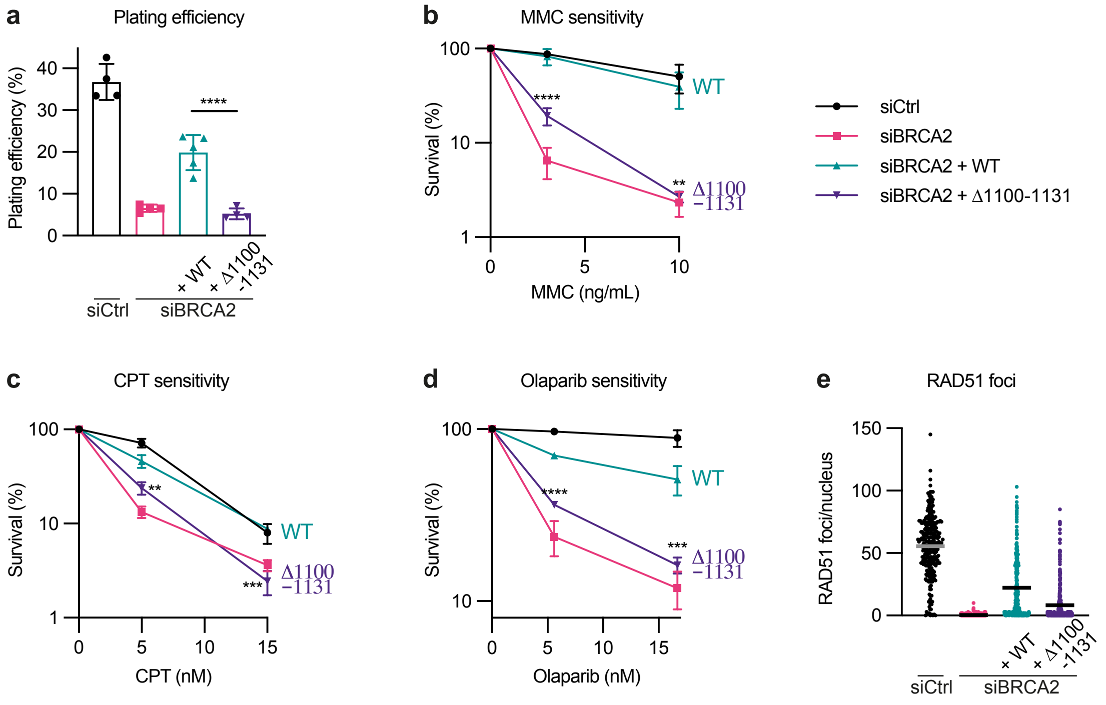

Supplementary Figure 3. Data related to Figure 2.

a-e. U2OS Flp-In T-REx parental cells or stably expressing siRNA resistant WT or Δ1100-1131 Venus-MBP-BRCA2 cDNAs were transfected with Ctrl or BRCA2 siRNA. The siCtrl, siBRCA2, and siBRCA2 + WT data are identical to Fig. 2b-f. **a-d**. Colony formation assays showing plating efficiency (**a**), MMC sensitivity (**b**), CPT sensitivity (**c**), and Olaparib sensitivity (**d**). Data are presented as means +/- standard deviations for three independent experiments except for in (a) and (b) where siBRCA2 + WT is n=5 and the remaining conditions are n=4. One-way ANOVA analyses with Dunnett's multiple comparison tests were performed to compare each condition to siBRCA2 + WT. **, p<0.1. ***, p<0.001. ****, p<0.0001. **e**. RAD51 nuclear foci in cells synchronized to S-phase with a thymidine block, released from the block and treated for 1 hour with MMC, and then allowed to recover for 8 hours before immunofluorescence microscopy. Each dot represents an individual nucleus, and means are indicated for 272 (siCtrl), 287 (siBRCA2), 239 (siBRCA2 + WT), or 306 (siBRCA2 + Δ1100-1131) nuclei. The experiment is a representative of three independent experiments.

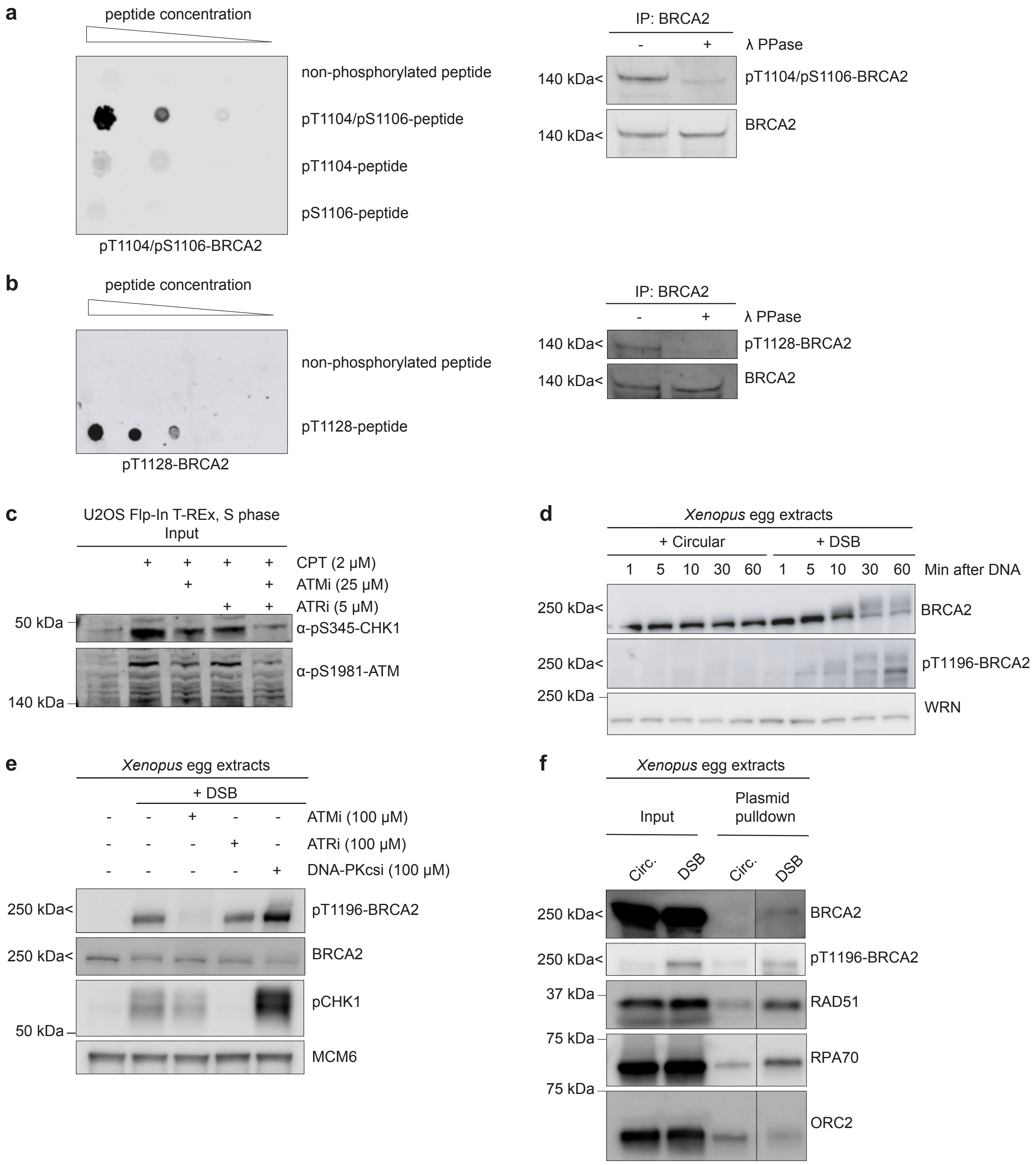

Supplementary Figure 4. Data related to Figure 3.

a. Validation of the phospho-specificity of the pT1104/pS1106-BRCA2 polyclonal antibody. Left: Dot blot of non-phosphorylated and phosphorylated versions of the BRCA2 peptide SNHNL(p)TP(p)SQKAEI. Right: Western blot of lambda phosphatase (λ PPase) treated immunoprecipitated BRCA2 from U2OS Flp-In T-REx cells synchronized to S-phase by a thymidine block, released for 1 hour, and then treated for 1 hour with 2 μM CPT. Representative of one experiment. IP, immunoprecipitation. **b**. Validation of the phospho-specificity of the pT1128-BRCA2 polyclonal antibody as in a. using the non-phosphorylated and phosphorylated BRCA2 peptide CQFEF(p)TQFRKPS for dot blotting. **c**. Western blot of cell extracts from U2OS Flp-In T-REx cells synchronized to S-phase by a thymidine block, released for 1 hour, and then treated for 1 hour with 2 μM CPT in presence or absence of ATM and ATR inhibitors. Representative of two independent experiments. **d**. Western blot of *Xenopus* egg extracts at the indicated times after addition of an intact circular or linearized DSB-containing plasmid representative of two independent experiments. **e**. Western blot of *Xenopus* egg extracts 1 hour after addition of a linearized DSB-containing plasmid in presence of ATM, ATR, or DNA-PKcs inhibitors representative of two independent experiments. **f**. Western blot of a plasmid pulldown with an intact circular (Circ.) or linearized DSB-containing plasmid from *Xenopus* egg extracts representative of three independent experiments. The order of the lanes has been rearranged as indicated by the black lines.

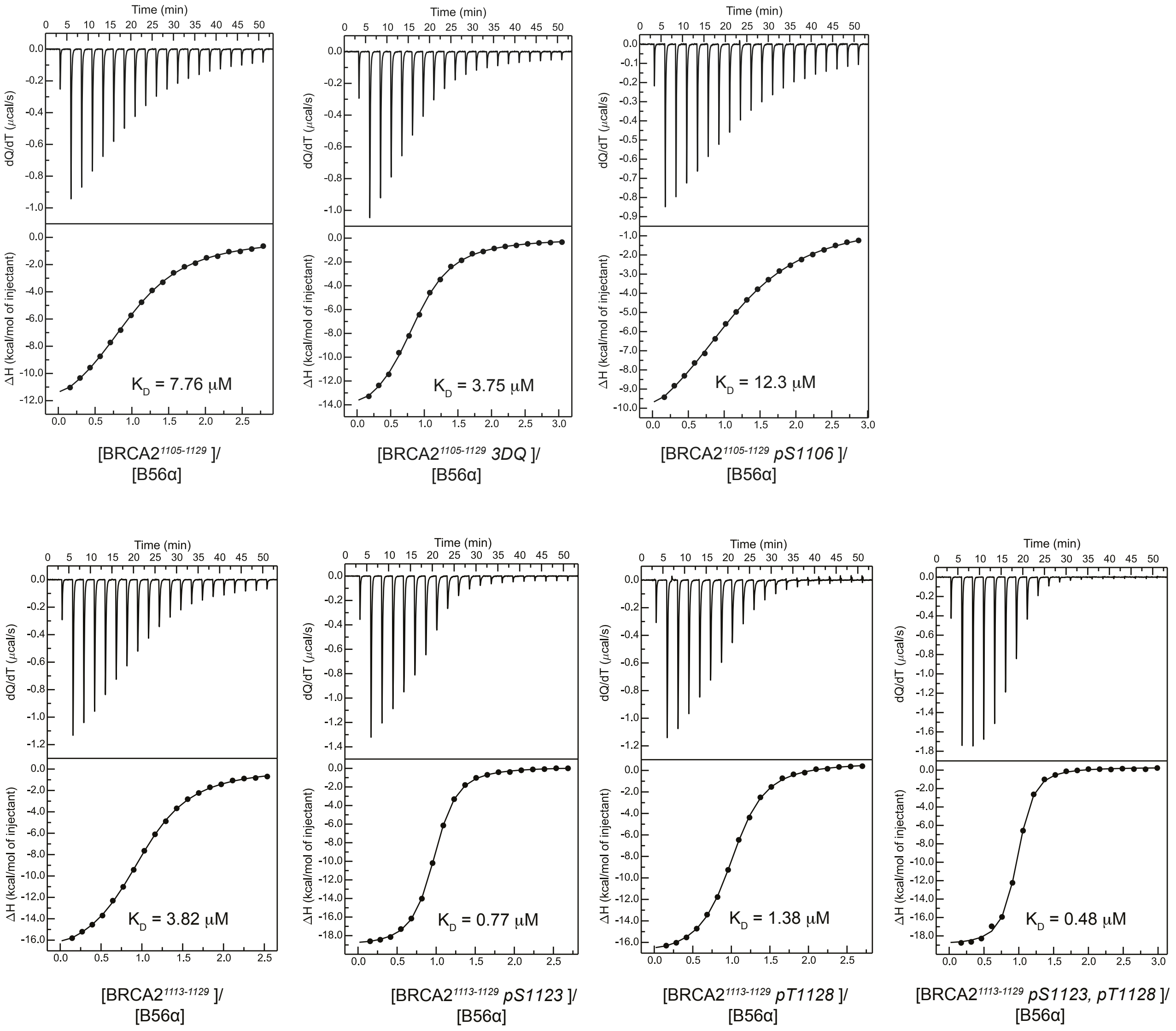

Supplementary Figure 5. Data related to Figure 3.

Isothermal titration calorimetry binding curves for the interactions between the indicated BRCA2 peptides and B56α.

a

B56α complex	K_D (μM)
BRCA2$^{1105-1129}$	7.76 ± 0.40
BRCA2$^{1105-1129}$ S1106R	2.93 ± 0.09
BRCA2$^{1105-1129}$ T1116P	6.92 ± 0.47
BRCA2$^{1105-1129}$ T1128I	7.43 ± 0.43

c

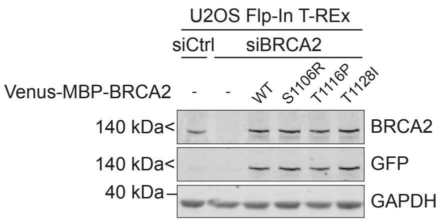

b

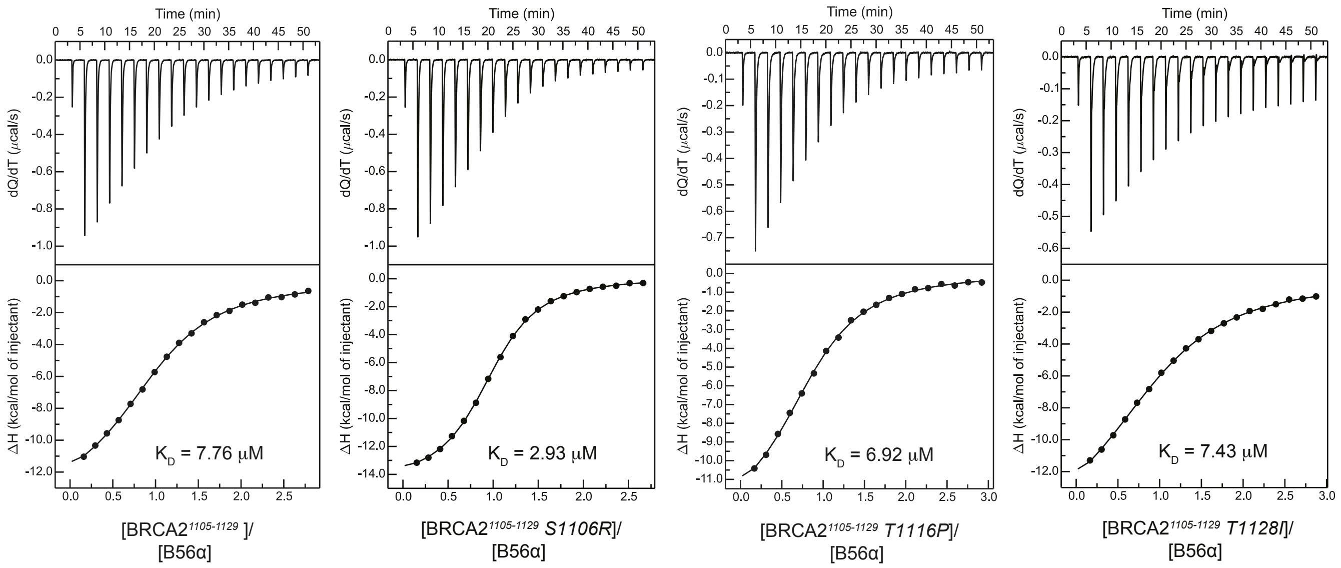

Supplementary Figure 6. Data related to Figure 4.

a-**b**. Dissociation constants (K_D) (**a**) and binding curves (**b**) for the interactions between the indicated BRCA2 peptides and B56α measured by isothermal titration calorimetry. The data for BRCA2$^{1105-1129}$ WT is identical to Fig. 3d and Supplementary Fig. 5. **c**. Western blot of cell extracts from U2OS Flp-In T-REx parental cells or stably expressing siRNA resistant WT, S1106R, T1116P, or T1128I Venus-MBP-BRCA2 cDNAs, which were transfected with Ctrl or BRCA2 siRNA. Representative of two independent experiments.

Supplementary Figure 7. Uncropped Western blots and gels.

1c

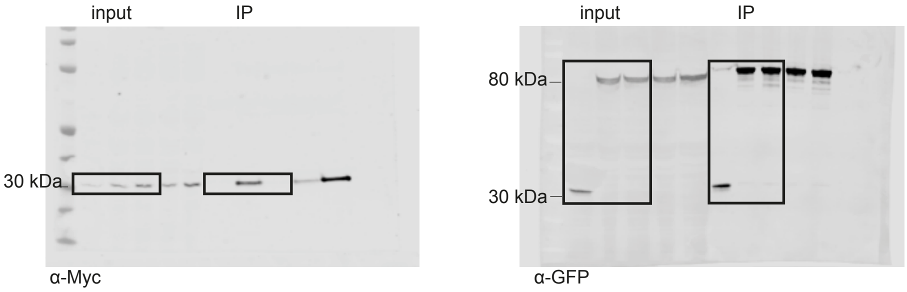

1d

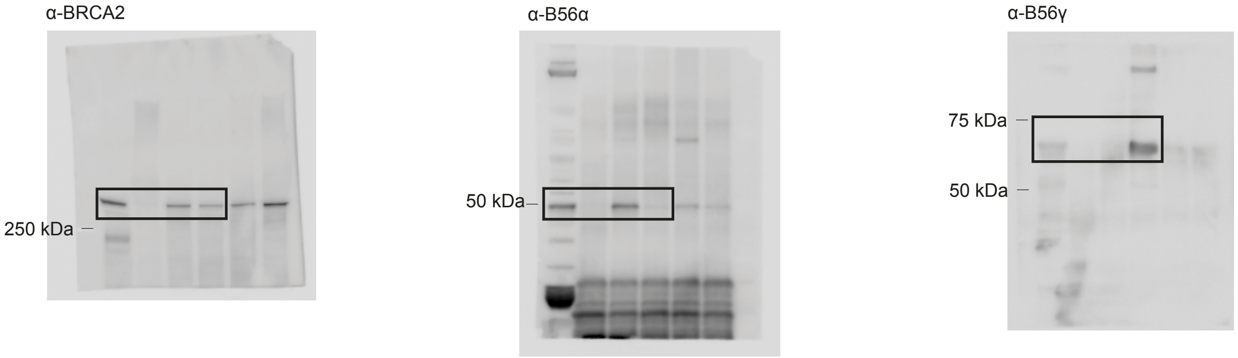

1f

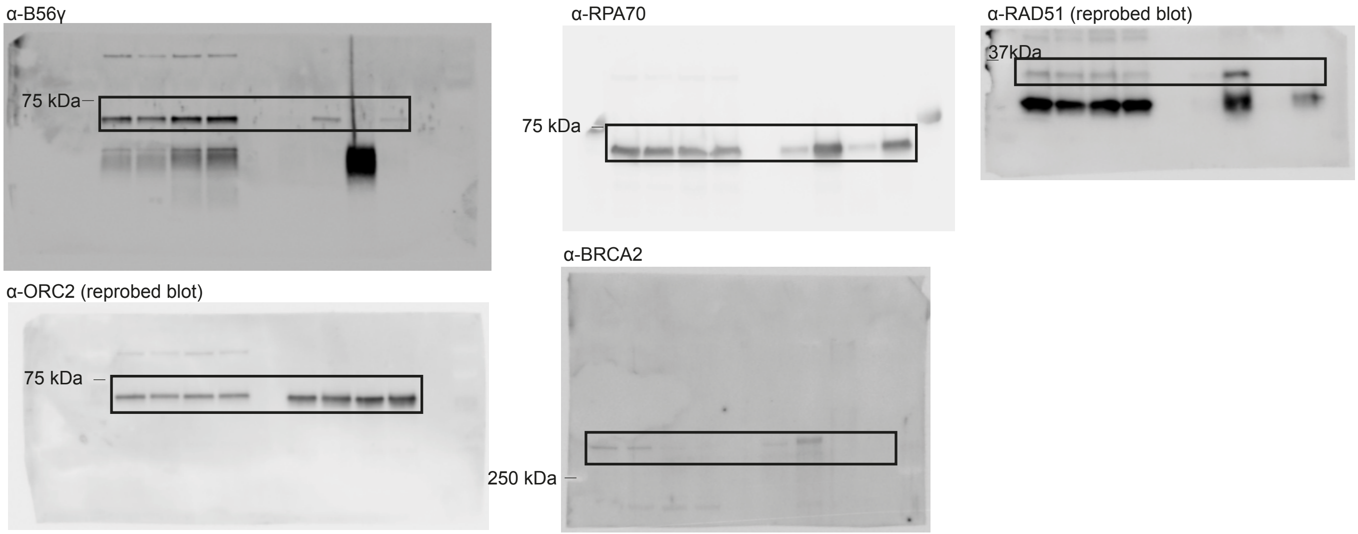

2g

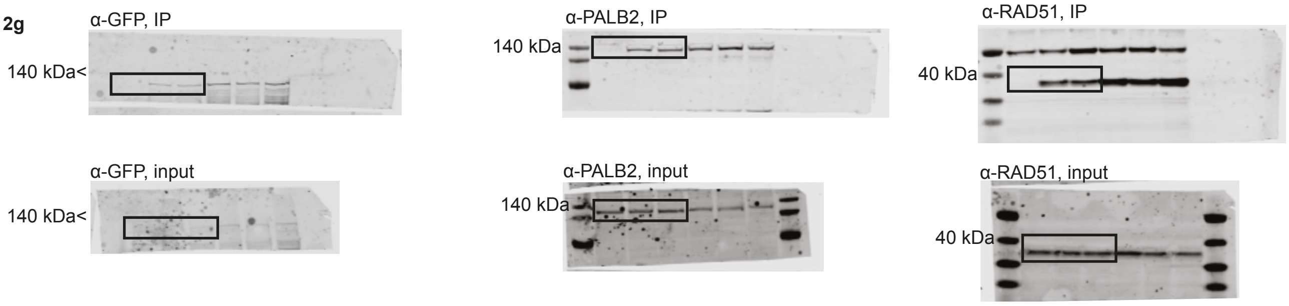

Supplementary Figure 7. Uncropped Western blots and gels.

3b

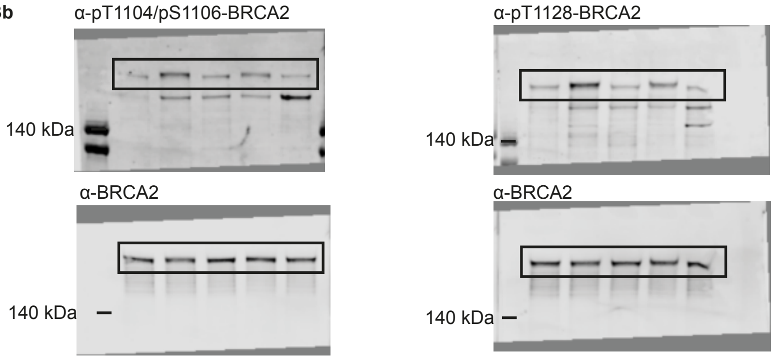

3c

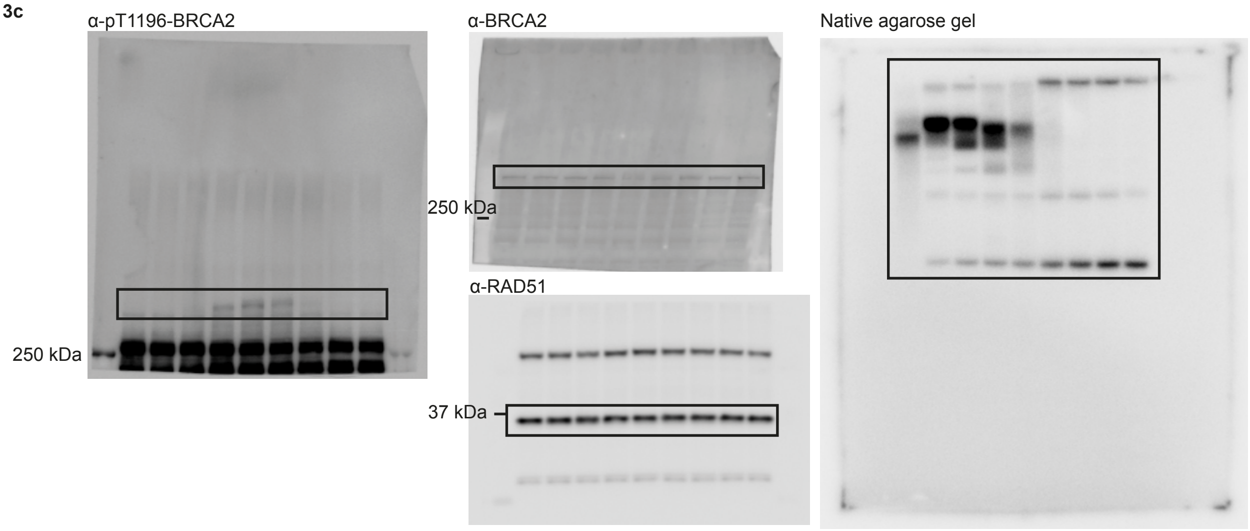

3e (identical to 1c, lanes rearranged)

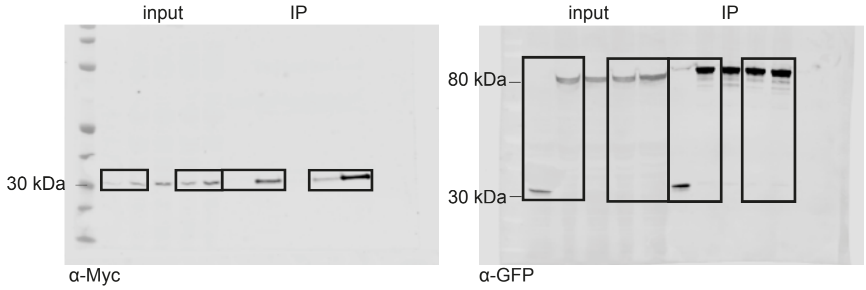

4b

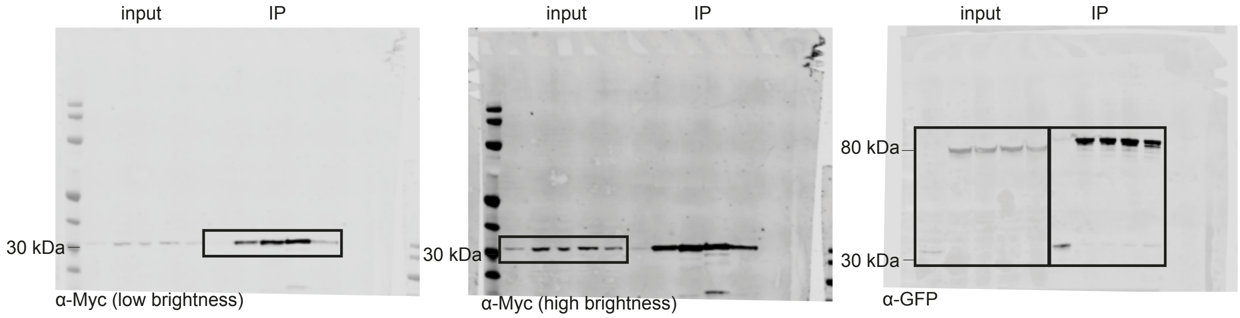

Supplementary Figure 7. Uncropped Western blots and gels.

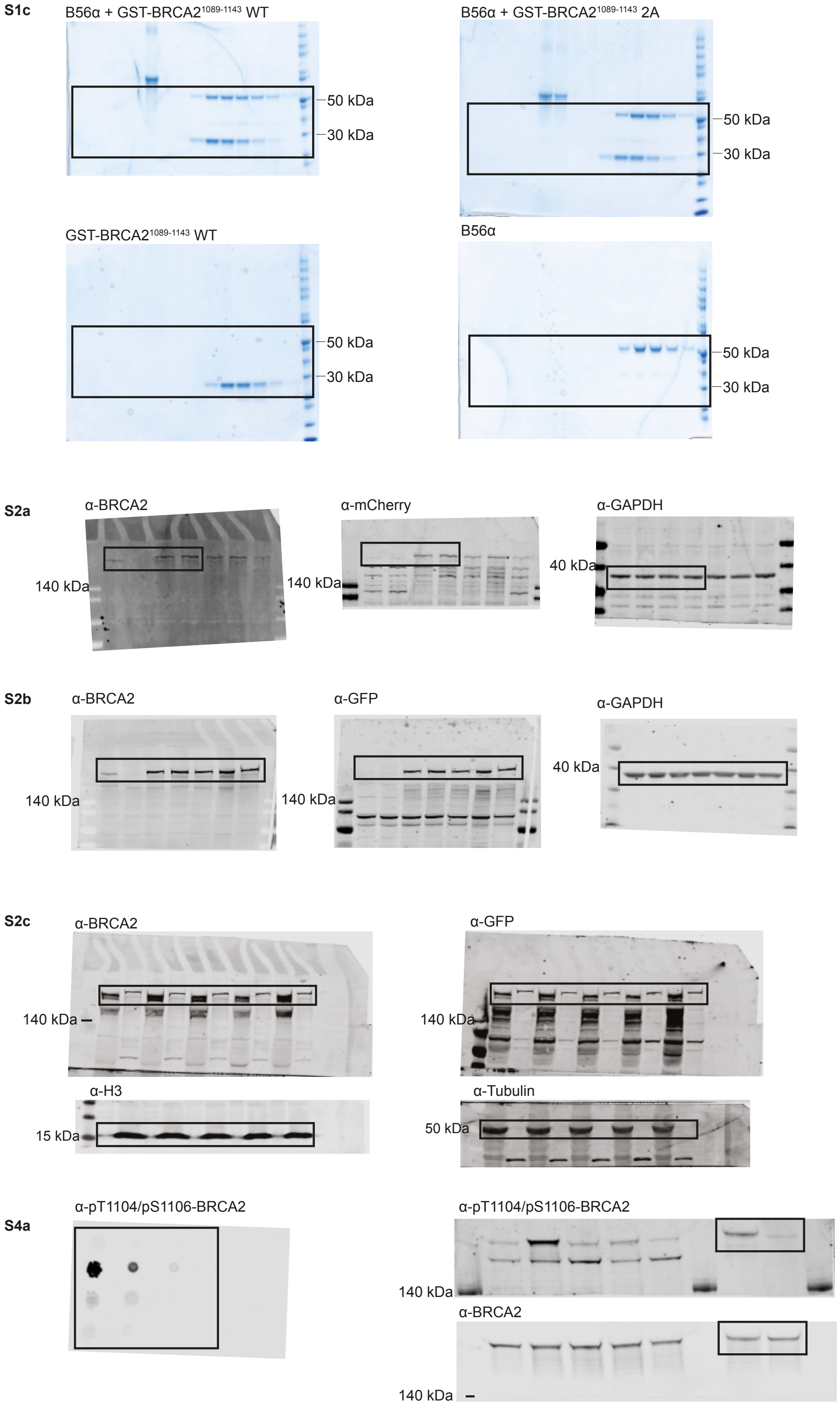

Supplementary Figure 7. Uncropped Western blots and gels.

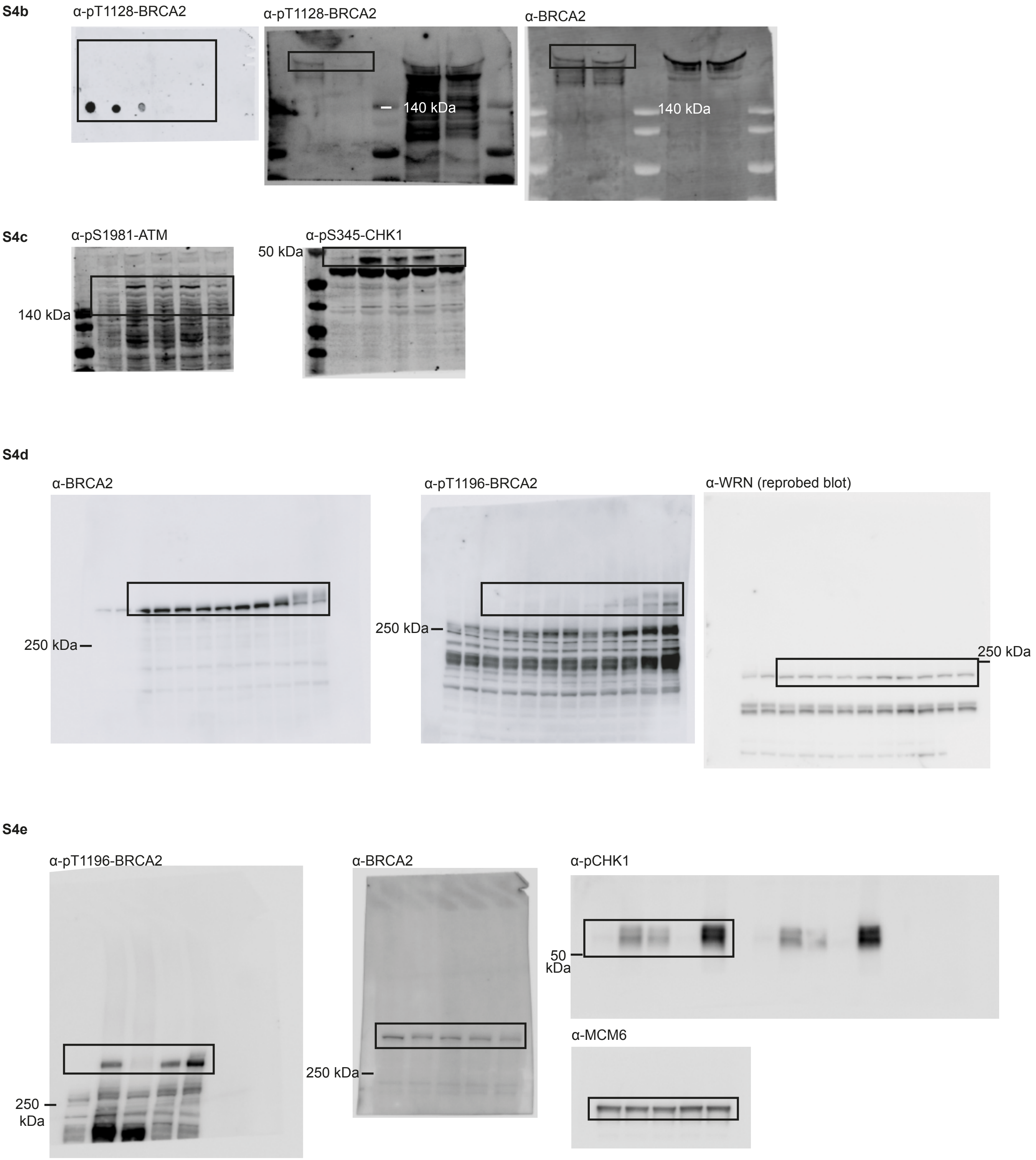

Supplementary Figure 7. Uncropped Western blots and gels.

S4f

α-BRCA2

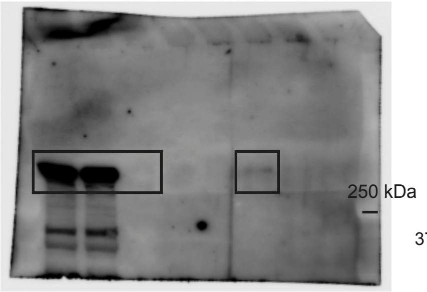

α-RAD51 (reprobed blot)

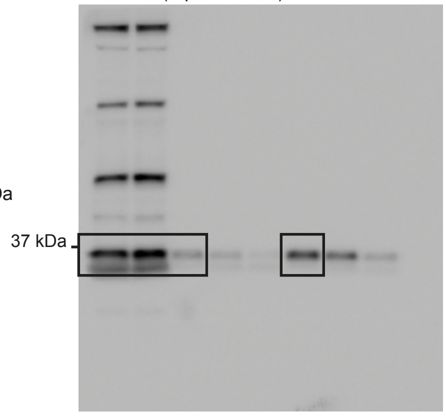

α-pT1196-BRCA2

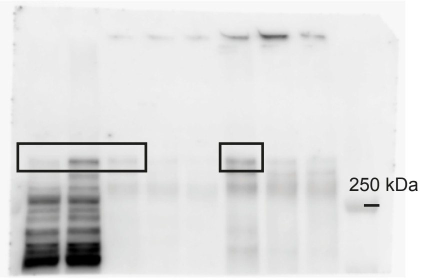

α-RPA70

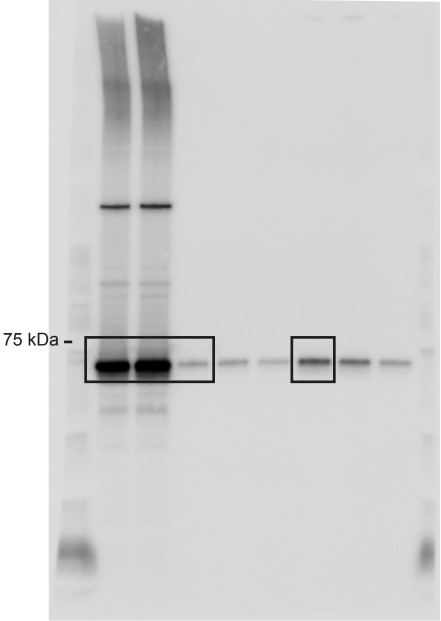

α-ORC2

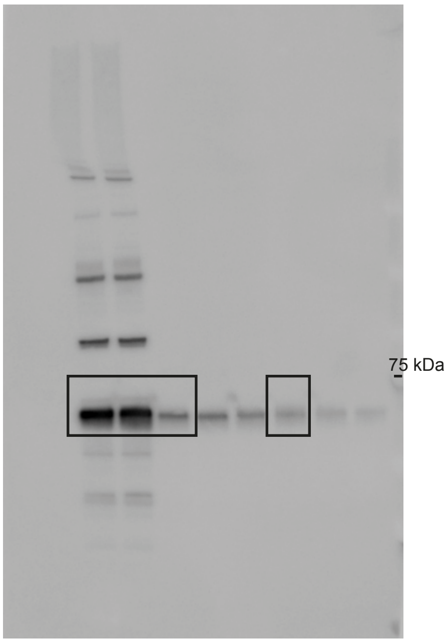

S6c

α-BRCA2

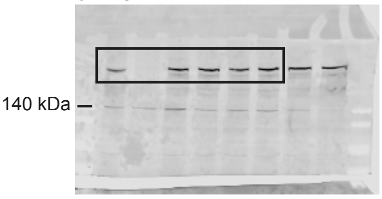

α-GFP

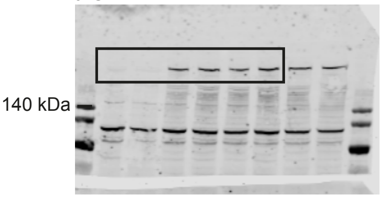

α-GAPDH

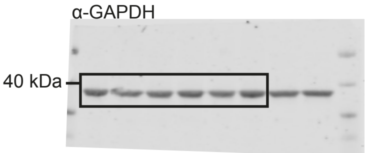

Supplementary Table 1. DNA oligos.

DNA oligos used in this study. QC, Quick change. F, Forward. R, Reverse.

Primer	Sequence (5'-3')
QC R for resistance to s2084 and s2085	GGAGAAGACATCATCTGGCCTGTATATCTTTCGCAATGAAAGAG
QC F for resistance to s2084 and s2085	CTCTTTCATTGCGAAAGATATACAGGCCAGATGATGTCTTCTCC
QC R to introduce L1114A-I1117A	CCTGATTCTTCTAATGCAGTAGAAGCTTCTGTAATTTCTGC
QC F to introduce L1114A-I1117A	GCAGAAATTACAGAAGCTTCTACTGCATTAGAAGAATCAGG
QC R to introduce S1106A	CTGCCTTTTGGGCAGGTGTTAAATTATGG
QC F to introduce S1106A	CCATAATTTAACACCTGCCCAAAAGGCAG
QC R to introduce S1123A	GCAAATTCAAACTGAGCTCCTGATTCTTC
QC F to introduce S1123A	GAAGAATCAGGAGCTCAGTTTGAATTTGC
QC R to introduce T1128A	GCTTTCTAAACTGAGCAAATTCAAACTG
QC F to introduce T1128A	CAGTTTGAATTTGCTCAGTTTAGAAAGC
F BRCA2 (to introduce Δ1100-113, 2 step)	CCCGGGGGTACCCCACCATGCCTATTGGATCCAAAGAGAGG
R BRCA2 1099 (to introduce Δ1100-1131, 2 step)	TATGTAGCTTGGCTTTGAATTAAAATCCTGCTTGG
F BRCA2 1132 (to introduce Δ1100-113, 2 step)	CAGGATTTTAATTCAAAGCCAAGCTACATATTGC
R BRCA2 (to introduce Δ1100-113, 2 step)	CCCGGGGCGGCCGCCCGATATATTTTTTAGTTGTAATTGTGTCC
QC R to introduce S1106D	CTGCCTTTTGGTCAGGTGTTAAATTATGG
QC F to introduce S1106D	CCATAATTTAACACCTGACCAAAAGGCAG
QC R to introduce S1123D	GAGTAAATTCAAACTGATCTCCTGATTCTTC
QC F to introduce S1123D	GAAGAATCAGGAGATCAGTTTGAATTTACTC
QC R to introduce T1128D	GCTTTCTAAACTGATCAAATTCAAACTG
QC F to introduce T1128D	CAGTTTGAATTTGATCAGTTTAGAAAGC
QC R to introduce S1106R	CAAACCATAATTTAACACCTAGGCAAAAGGCAGAAATTACAGAAC
QC F to introduce S1106R	GTTCTGTAATTTCTGCCTTTTGCCTAGGTGTTAAATTATGGTTTG
QC R to introduce T1116P	AGGCAGAAATTACAGAACTTTCTCCTATATTAGAAGAATCAGGAAGT
QC F to introduce T1116P	ACTTCCTGATTCTTCTAATATAGGAGAAAGTTCTGTAATTTCTGCCT
QC R to introduce T1128I	AGAATCAGGAAGTCAGTTTGAATTTATTCAGTTTAGAAAGCCAAGC
QC F to introduce T1128I	GCTTGGCTTTCTAAACTGAATAAATTCAAACTGACTTCCTGATTCT
R to create BamHI-Myc-BRCA2$^{1001-1255}$-NotI	CCCGGGGCGGCCGCTCATACCTCTGCAGAAGTTTCC
F to create BamHI-Myc-BRCA2$^{1001-1255}$-NotI	CCCGGGGGATCCCCACCATGGAACAAAAGTTGATCAGCGA<br>GGAGGACCTGTCAAATCACAGTT TTGGAGG
R to create BamHI-BRCA2$^{1001-1255}$-NotI	CGATGCGGCCGCTTATTCCTCACTAATATTCTC
F to create BamHI-BRCA2$^{1001-1255}$-NotI	CGATGGATCCATCTCCTTGAATATAGAT

Supplementary Table 2. Isothermal titration calorimetry data.

Affinities and thermodynamic values of B56α/BRCA2 peptide binding events inferred from ITC measurements performed at 25°C. Gibbs free energy (ΔG), enthalpy (ΔH), entropy (-TΔS), equilibrium dissociation constant (K_D) and reaction stoichiometry (n) are shown. The affinity is defined by the Gibbs energy for binding $\Delta G = -RT \ln K_A = RT \ln K_D$. The errors represent the standard error of the fitting. Related to Figure 1, 3, and 4.

B56α complex	Peptide sequence	ΔG (kcal mol^{-1})	ΔH (kcal mol^{-1})	-TΔS (kcal mol^{-1})	K_D (μM)	n
B56α/BRCA2$^{1108\text{-}1126}$	WKAEITELSTILEESGSQFE	-7.21	-14.9 ± 0.20	7.69	5.17 ± 0.25	1.02 ± 0.01
B56α/BRCA2$^{1108\text{-}1126}$ 2A	WKAEITEASTALEESGSQFE	No binding				
B56α/BRCA2$^{1105\text{-}1129}$	PSQKAEITELSTILEESGSQFEFTQW	-6.97	-13.8 ± 0.25	6.87	7.76 ± 0.40	0.99 ± 0.01
B56α/BRCA2$^{1105\text{-}1129}$ pS1106	P(pS)QKAEITELSTILEESGSQFEFTQW	-6.70	-12.2 ± 0.30	5.50	12.3 ± 0.76	1.19 ± 0.01
B56α/BRCA2$^{1105\text{-}1129}$ 3DQ	PDQKAEITELSTILEESGDQFEFDQW	-7.40	-15.4 ± 0.24	8.02	3.75 ± 0.22	0.86 ± 0.01
B56α/BRCA2$^{1105\text{-}1129}$ S1106R	PRQKAEITELSTILEESGSQFEFTQW	-7.55	-14.4 ± 0.10	6.89	2.93 ± 0.09	0.95 ± 0.00
B56α/BRCA2$^{1105\text{-}1129}$ T1116P	PSQKAEITELSPILEESGSQFEFTQW	-7.04	-13.4 ± 0.32	6.35	6.92 ± 0.47	0.85 ± 0.01
B56α/BRCA2$^{1105\text{-}1129}$ T1128I	PSQKAEITELSTILEESGSQFEFIQW	-7.00	-16.2 ± 0.41	9.24	7.43 ± 0.43	1.01 ± 0.00
B56α/BRCA2$^{1113\text{-}1129}$	WELSTILEESGSQFEFTQ	-7.39	-18.0 ± 0.15	10.60	3.82 ± 0.12	1.01 ± 0.00
B56α/BRCA2$^{1113\text{-}1129}$ pS1123	WELSTILEESG(pS)QFEFTQ	-8.34	-19.3 ± 0.09	11.00	0.77 ± 0.03	0.92 ± 0.00
B56α/BRCA2$^{1113\text{-}1129}$ pT1128	WELSTILEESGSQFEF(pT)Q	-7.99	-17.7 ± 0.13	9.66	1.38 ± 0.04	0.94 ± 0.00
B56α/BRCA2$^{1113\text{-}1129}$ pS1123, pT1128	WELSTILEESG(pS)QFEF(pT)Q	-8.62	-19.3 ± 0.14	10.70	0.48 ± 0.03	0.91 ± 0.00
