# Supplementary material for: A complex of BRCA2 and PP2A-B56 is required for DNA repair by homologous recombination
Source: Nat Commun. 2021 Sep 30;12:5748. doi: 10.1038/s41467-021-26079-0 (PMC8484605; doi:10.1038/s41467-021-26079-0)
Supplement: Supplementary file 3 — Description of Additional Supplementary Files [file 41467_2021_26079_MOESM3_ESM.pdf]

Description of Additional Supplementary Files

File Name: Supplementary Data 1
Description: **Conservation of the B56 binding motif in BRCA2.**
Clustal Omega multiple sequence alignment of 190 vertebrate BRCA2 protein sequences. The region around the B56 binding motif is shown. Related to Figure 1.

File Name: Supplementary Data 2
Description: **Mass spectrometry data.**
Related to Supplementary Fig. 1a: Mass spectrometry data of proteins copurifying with Venus or Venus-BRCA21001-1255. HeLa cells were transiently transfected with constructs of Venus or Venus-BRCA21001-1255, synchronized to S phase with a thymidine block, released for 2 hours, and then treated for 2 hours with 100 nM CPT prior to cell harvest, GFP-trap immunoprecipitation, and mass spectrometry analysis. Statistical analysis was carried out by two-tailed Student's t-test of three technical replicates.
