# Supplementary material for: A complex of BRCA2 and PP2A-B56 is required for DNA repair by homologous recombination
Source: Nat Commun. 2021 Sep 30;12:5748. doi: 10.1038/s41467-021-26079-0 (PMC8484605; doi:10.1038/s41467-021-26079-0)
Supplement: Supplementary file 6 — Reporting Summary [file 41467_2021_26079_MOESM6_ESM.pdf]

nature portfolio

Corresponding author(s): Jakob Nilsson, Michael Lisby, Vibe H. Oestergaard

Last updated by author(s): Sep 9, 2021

Reporting Summary

Nature Portfolio wishes to improve the reproducibility of the work that we publish. This form provides structure for consistency and transparency in reporting. For further information on Nature Portfolio policies, see our Editorial Policies and the Editorial Policy Checklist.

Statistics

For all statistical analyses, confirm that the following items are present in the figure legend, table legend, main text, or Methods section.

n/a	Confirmed	
☐	☒	The exact sample size (*n*) for each experimental group/condition, given as a discrete number and unit of measurement
☐	☒	A statement on whether measurements were taken from distinct samples or whether the same sample was measured repeatedly
☐	☒	The statistical test(s) used AND whether they are one- or two-sided<br>*Only common tests should be described solely by name; describe more complex techniques in the Methods section.*
☒	☐	A description of all covariates tested
☐	☒	A description of any assumptions or corrections, such as tests of normality and adjustment for multiple comparisons
☐	☒	A full description of the statistical parameters including central tendency (e.g. means) or other basic estimates (e.g. regression coefficient) AND variation (e.g. standard deviation) or associated estimates of uncertainty (e.g. confidence intervals)
☐	☒	For null hypothesis testing, the test statistic (e.g. *F*, *t*, *r*) with confidence intervals, effect sizes, degrees of freedom and *P* value noted<br>*Give P values as exact values whenever suitable.*
☒	☐	For Bayesian analysis, information on the choice of priors and Markov chain Monte Carlo settings
☒	☐	For hierarchical and complex designs, identification of the appropriate level for tests and full reporting of outcomes
☒	☐	Estimates of effect sizes (e.g. Cohen's *d*, Pearson's *r*), indicating how they were calculated

Our web collection on statistics for biologists contains articles on many of the points above.

Software and code

Policy information about availability of computer code

Data collection	ImageStudio v. 3.1.4. (LI-COR) and ImageQuant LAS4000 v. 1.2. (GE healthcare) were used for Western blot acquisition. GelCount v. 1.3.04. (Oxford Optronix) was used to image and quantify clonogenic survival assays. DeltaVision SoftWoRx v. 7.0.0. (GE healthcare) was used for immunofluorescence microscopy acquisition. BD FACSDiva v. 9 (BD Biosciences) was used for flow cytometry acquisition. All ITC experiments were performed on an Auto-iTC200 instrument (Auto-iTC200 v. 1.1.1.0, iTC200 v. 1.26.4, Origin v. 7.0552 SR4).
Data analysis	PRISM v. 9.1.1. (GraphPad) was used for data plotting and statistics. FCS Express v. 7.04. (De Novo Software) was used to analyse Flow Cytometry data. ImageStudioLite v. 5.2.5. (LI-COR) was used for Western blot quantification. DeltaVision SoftWoRx v. 5.0.0. (GE healthcare) was used for analyzing immunofluorescence microscopy data. MicroCal PEAQ-ITC v. 1.1.0.1262 (Malvern Panalytical) was used for analyzing ITC data. Extinction coefficients were computed from the corresponding sequences by the ProtParam program (http://web.expasy.org/protparam/). Mass spectrometry raw data were searched using COMET (release version 2014.01) (http://comet-ms.sourceforge.net) in high resolution mode against a target-decoy (reversed) version of the human proteome sequence database (UniProt downloaded 2/2020, (https://www.uniprot.org)). Mass spectrometry data was analyzed in Perseus v. 1.6.14.0.(MaxQuant). Clustal omega multiple sequence alignment (https://www.ebi.ac.uk/Tools/msa/clustalo/) were performed on vertebrate BRCA2 protein sequences downloaded from the NCBI protein database (https://www.ncbi.nlm.nih.gov/protein/). PyMOL Molecular Graphics System v. 2.3.4. was used to generate Fig. 1a.

For manuscripts utilizing custom algorithms or software that are central to the research but not yet described in published literature, software must be made available to editors and reviewers. We strongly encourage code deposition in a community repository (e.g. GitHub). See the Nature Portfolio guidelines for submitting code & software for further information.

Data

Policy information about availability of data

All manuscripts must include a data availability statement. This statement should provide the following information, where applicable:

- Accession codes, unique identifiers, or web links for publicly available datasets
- A description of any restrictions on data availability
- For clinical datasets or third party data, please ensure that the statement adheres to our policy

Vertebrate BRCA2 protein sequences used for Clustal Omega multiple sequence alignment were downloaded from the NCBI protein database (https://www.ncbi.nlm.nih.gov/protein/). Evolution tree was generated using the TimeTree database (timetree.org). The human proteome sequence database used for mass spectrometry analysis were downloaded (2/2020) from UniProt (https://www.uniprot.org). The mass spectrometry data generated in this work have been deposited to ProteomeXchange under accession code PXD027574 (http://proteomecentral.proteomexchange.org/cgi/GetDataset?ID=PXD027574) and MassIVE under accession code MSV000087884 (https://massive.ucsd.edu/ProteoSAFe/private-dataset.jsp?task=9725acdfd8084e2e8ebcaea599cee084). The DR-GFP reporter assay data, colony survival assay data, and RAD51 foci data generated in this study are provided in the source data. Source data are provided with this paper.

Field-specific reporting

Please select the one below that is the best fit for your research. If you are not sure, read the appropriate sections before making your selection.

- [x] Life sciences
- [ ] Behavioural & social sciences
- [ ] Ecological, evolutionary & environmental sciences

For a reference copy of the document with all sections, see nature.com/documents/nr-reporting-summary-flat.pdf

Life sciences study design

All studies must disclose on these points even when the disclosure is negative.

Sample size	No statistical method was used to predetermine sample size. For RAD51 foci experiments, minimum 150 individual cells were analyzed per condition and the experiment was performed three times independently. The data from the three individual experiments is provided in the source data and one experiment is shown in the Fig. 2f and 3j, and Supplementary Fig. 3e. For all other experiments, sample size is not applicable.
Data exclusions	No predetermined exclusion criteria was established. We have excluded data where the tissue culture plate was contaminated. This is the case for the siBRCA2 + 3AQ condition for one of the experiments in Fig. 3h.
Replication	The experimental findings were reliably reproduced and in many instances confirmed by multiple experimental approaches. All colony survival assays and RAD51 foci formation experiments were independently replicated at least three times except for Fig. 3h, where one replicate was taken out for the siBRCA2 + 3AQ condition due to contamination in the tissue culture plate. The mass spectrometry analysis in Supplementary Fig. 1a is based on a technical triplicate experiment while Supplementary Fig. 1d is based on three independent experiments. All immunoprecipitation experiments were independently replicated minimum two times with similar results except validation of phospho-antibodies in Supplementary Fig 4a and b, which were performed once. All Xenopus egg extract experiments were independently replicated minimum two times with similar results. Direct biochemical binding experiments including size exclusion chromatography and ITC were performed once. Number of replicates for all experiments are indicated in the figure legends.
Randomization	Randomization was not possible for the types of experiments included in this work.
Blinding	Quantification of colony formation was done automatically and quantification of RAD51 foci semi-automatically. Therefore no blinding was required for these experiments. For biochemical analyses, all samples were treated in the same way, and therefore blinding is not relevant.

Reporting for specific materials, systems and methods

We require information from authors about some types of materials, experimental systems and methods used in many studies. Here, indicate whether each material, system or method listed is relevant to your study. If you are not sure if a list item applies to your research, read the appropriate section before selecting a response.

Materials & experimental systems

n/a	Involved in the study	
☐	☒	Antibodies
☐	☒	Eukaryotic cell lines
☒	☐	Palaeontology and archaeology
☐	☒	Animals and other organisms
☒	☐	Human research participants
☒	☐	Clinical data
☒	☐	Dual use research of concern

Methods

n/a	Involved in the study	
☒	☐	ChIP-seq
☐	☒	Flow cytometry
☒	☐	MRI-based neuroimaging

Antibodies

Antibodies used

The following antibodies were used for Western blot analysis or immunofluorescence microscopy staining in the indicated dilutions unless otherwise stated:
BRCA2: Millipore Cat# OP95, RRID:AB_2067762, 1:1000.
RAD51: Bio Academia Cat# 70-001, 1:1000.
RFP (mCherry): MBL International Cat# PM005, RRID:AB_591279, 1:1000.
Myc: Santa Cruz Biotechnology Cat# sc-40, RRID:AB_627268, 1:750.
PALB2: Bethyl Cat# A301-246A, RRID:AB_890607, 1:1000.
GAPDH: Santa Cruz Biotechnology Cat# sc-25778, RRID:AB_10167668, 1:5000.
Tubulin: Abcam Cat# ab6160, RRID:AB_305328, 1:5000.
Histone 3: Abcam Cat# ab1791, RRID:AB_302613, 1:1000.
pS345-CHK1: Cell Signaling Technology Cat# 2341, RRID:AB_330023, 1:1000.
pS1981-ATM: Millipore Cat# MAB3806, RRID:AB_569379, 1:2000.
PP2A-C: Millipore Cat# 05-421, RRID:AB_309726, 1:1000.
GFP (Venus): serum produced by Moravian, affinity purified against full-length GFP, 1:5000.
pT1104/pS1106-BRCA2: raised in rabbits using the phosphorylated peptide of BRCA2: SNHNL(pT)P(pS)QKAEI for immunization, affinity purification, and validation (21st Century Biochemicals). Dilution: 1:500.
pT1128-BRCA2: raised in rabbits using the phosphorylated peptide of BRCA2: CQFEF(pT)QFRKPS for immunization, affinity purification, and validation (Moravian). Dilution: 1:500.
Goat-anti-rabbit IgG AlexaFluor 546 nm: Life Technologies Cat# A-11010, 1:1000.

Xenopus specific antibodies:
BRCA2 (for Western blot analyses): Described previously in Long et al., 2014. Dilution: 1:5000.
BRCA2 (for Western blot analysis in Supplementary Fig. 4d): Described previously in Kolinjivadi et al., 2017. Dilution: 1:500.
BRCA2 (for immunodepletion): Raised in rabbits against the following peptide: Ac-KPHIKEDQNEPESNSEYC-amide by New England Peptide as described previously (Kolinjivadi et al., 2017).
B56 alpha/beta raised in rabbits against the following peptide: MSAISAAEKVDGFTRKSVRK by Peptide Speciality Laboratories GmbH. Dilution: 1:1000.
B56 gamma: raised in rabbits against the following peptide: MPNKNKKDKEPPKAGKSGKS by Peptide Speciality Laboratories GmbH. Dilution: 1:500.
RAD51: Described previously in Long et al., 2011. Dilution: 1:2500.
RPA70: Described previously in Gallina et al., 2021. Dilution: 1:2500.
ORC2: Described previously in Fang & Newport, 1993. Dilution: 1:2500.
WRN: raised in rabbits against the following peptide: H2N-MTSLQRKLPEWMSVKC-amide by New England Peptide. Dilution: 1:2500.
MCM6: Described previously in Larsen et al., 2019. Dilution: 1:2500.

Validation

BRCA2 (Millipore Cat# OP95): Validation found at https://www.merckmillipore.com/DK/en/product/Anti-BRCA2-Ab-1-Mouse-mAb-2B,EMD_BIO-OP95. Additionally, we validated this antibody by Western blot analysis of cell extract where BRCA2 had been depleted by siRNA-mediated knockdown, observing that the band disappeared after depletion. Moreover, the antibody recognizes exogenously expressed venus-MBP-BRCA2 from cell extracts analyzed by Western blotting.
RAD51 (Bio Academia Cat# 70-001): Validation found at https://www.bioacademia.co.jp/en/html/upload/save_image/E70-012%20anti-Rad51%20(human)antibody%20rabbit%20polyclonal.pdf. Additionally, we validated this antibody by immunofluorescence microscopy of cells where BRCA2 had been depleted by siRNA-mediated knockdown, observing that the RAD51 foci dissappeared after BRCA2 depletion.
RFP (mCherry) (MBL International Cat# PM005): Validation found at https://www.mblintl.com/products/pm005/.
Myc (Santa Cruz Biotechnology Cat# sc-40): Validation found at https://www.scbt.com/p/c-myc-antibody-9e10.
PALB2 (Bethyl Cat# A301-246A): Validation found at https://www.bethyl.com/product/A301-246A/PALB2+Antibody.
GAPDH (Santa Cruz Biotechnology Cat# sc-25778): Validation found at https://www.scbt.com/p/gapdh-antibody-fl-335.
Tubulin (Abcam Cat# ab6160): Validation found at https://www.abcam.com/tubulin-antibody-yl12-loading-control-ab6160.html.
Histone 3 (Abcam Cat# ab1791): Validation found at https://www.abcam.com/histone-h3-antibody-nuclear-marker-and-chip-grade-ab1791.html.
pS345-CHK1 (Cell Signaling Technology Cat# 2341): Validation found at https://www.cellsignal.com/products/primary-antibodies/phospho-chk1-ser345-antibody/2341.
pS1981-ATM (Millipore Cat# MAB3806): Validation found at https://www.merckmillipore.com/DK/en/product/Anti-ATM-phosphoSer1981-Antibody-clone-10H11.E12,MM_NF-MAB3806.
PP2A-C (Millipore Cat# 05-421): Validation found at https://www.merckmillipore.com/DK/en/product/Anti-PP2A-Antibody-C-subunit-clone-1D6,MM_NF-05-421.
GFP (Venus): Validated by Western blot analysis by its capacity to recognize GFP-trapped contructs of Venus (YFP-variant).
pT1104/pS1106-BRCA2: Validated by dot blot analysis using phosphorylated and non-phosphorylated peptides and by Western blot analysis of immunoprecipitated BRCA2 from U2OS Flp-In T-REx cells treated with lambda phosphatase.
pT1128-BRCA2: Validated by dot blot analysis using phosphorylated and non-phosphorylated peptides and by Western blot analysis of immunoprecipitated BRCA2 from U2OS Flp-In T-REx cells treated with lambda phosphatase.
Goat-anti-rabbit IgG AlexaFluor 546 nm (Life Technologies Cat# A-11010): Validation found at https://www.thermofisher.com/antibody/product/Goat-anti-Rabbit-IgG-H-L-Cross-Adsorbed-Secondary-Antibody-Polyclonal/A-11010

Xenopus specific antibodies:
BRCA2 (for Western blot analyses): Validated in Long et al., 2014.
BRCA2 (for Western blot analysis in Supplementary Fig. 4d): Validated in Kolinjivadi et al., 2017.

BRCA2 (for immunodepletion): Validated by capacity to immunoprecipitate and immunodeplete BRCA2 from Xenopus egg extracts as analyzed by Western blot with an existing validated BRCA2 antibody (Long et al., 2014).
B56 alpha/beta: Validated by Western blot analysis of in vitro transcribed and translated flag-tagged B56 and B55δ proteins.
B56 gamma: Validated by Western blot analysis of in vitro transcribed and translated flag-tagged B56 and B55δ proteins.
RAD51: Validated in Long et al., 2011.
RPA70: Validated in Gallina et al., 2021.
ORC2: Validated in Fang & Newport, 1993.
WRN: Validated by capacity to immunoprecipitate a band of the right size and immunodeplete a band of the right size from Xenopus egg extracts analyzed by Western blot.
MCM6: Validated in Larsen et al., 2019.

Eukaryotic cell lines

Policy information about cell lines

Cell line source(s)	HeLa cells were acquired from ATCC. The U2OS Flp-In T-Rex cell line was a kind gift from Helen Piwnica-Worms. The HeLa Flp-In-T-Rex cell line was a kind gift from Stephen Taylor. The HeLa DR-GFP Flp-In cell line was a kind gift from Jeffrey Parvin. All stable cell lines generated for this study from these parental cells are available upon request.
Authentication	No authentication was performed.
Mycoplasma contamination	The cell lines were not tested for mycoplasma contamination.
Commonly misidentified lines (See ICLAC register)	No common misidentified cell lines were used.

Animals and other organisms

Policy information about studies involving animals; ARRIVE guidelines recommended for reporting animal research

Laboratory animals	Mature wild type female Xenopus laevis frogs (>9cm) were used for egg extract preparation (LM00535MX, Nasco, USA)<br>Mature wild type male Xenopus laevis (7.5-9 cm) were used for sperm chromatin isolation (LM00715MX, Nasco, USA).<br>Frogs were >2 years old.
Wild animals	The study did not include wild animals.
Field-collected samples	The study did not involve samples collected from the field.
Ethics oversight	All experiments involving animals were approved by the Danish Animal Experiments Inspectorate and are conform to relevant regulatory standards and European guidelines.

Note that full information on the approval of the study protocol must also be provided in the manuscript.

Flow Cytometry

Plots

Confirm that:

- [x] The axis labels state the marker and fluorochrome used (e.g. CD4-FITC).
- [x] The axis scales are clearly visible. Include numbers along axes only for bottom left plot of group (a 'group' is an analysis of identical markers).
- [x] All plots are contour plots with outliers or pseudocolor plots.
- [x] A numerical value for number of cells or percentage (with statistics) is provided.

Methodology

Sample preparation	To analyze HR efficiency for full-length BRCA2 constructs (Fig. 2a), HeLa DR-GFP Flp-In cells parental or stably expressing siRNA resistant mCherry-MBP-BRCA2 were transfected with Ctrl or BRCA2 siRNA . The second siRNA transfection was combined with transient transfection with or without an I-SceI-encoding plasmid. After 48 hours, samples were prepared for flow cytometry. For the B56 inhibitor experiment (Supplementary Fig. 2d), HeLa DR-GFP Flp-In cells were transiently transfected with a plasmid encoding an mCherry-tagged version of the B56 substrate inhibitor or a control version of the inhibitor (described further in the manuscript) either with or without an I-SceI-encoding plasmid. After 48 hours, cells were prepared for flow cytometry. For sample preparation, cells were trypsinized, dissolved in 2% BSA in PBS, stained with 1 ug/mL DAPI, and filtered through a 35 um cell strainer.
Instrument	BD LSRFortessa (BD biosciences).
Software	Image acquisition was performed with BD FACSDiva v. 9 (BD biosciences). Data analysis was performed in FCS express v. 7.04. (De Novo Software). Graphs were constructed in PRISM v. 9.1.1. (GraphPad).

Cell population abundance	During acquisition, a preliminary gating strategy was applied to record 10000 living cells (DAPI negative, P3).
Gating strategy	First, a FSC-A/SSC-A plot was used to select cells and exclude debris based on size and granularity (P1). Second, a FSC-W/FSC-H plot was used to exclude doublets (P2). Third, a FSC-A/DAPI-A plot was used to include only the DAPI negative (and therefore living) cells (P3). For the BRCA2 knockdown and complementation experiment (Fig. 2a), a FSC-A/GFP-A plot was used to gate for the high GFP-expressing cells where homologous recombination has taken place. For the B56 inhibitor experiment (Supplementary Fig. 2d), a mCherry-A/GFP-A plot combined with a quadrant gating strategy was used to divide the population into mCherry positive/negative and GFP positive/negative fractions. The gates were valid in single-color and no-color control cells.

☒ Tick this box to confirm that a figure exemplifying the gating strategy is provided in the Supplementary Information.
